# Internal and External Motivations and Risk Perception toward COVID-19 Vaccination in Adolescents in the U.S.

**DOI:** 10.3390/vaccines10050697

**Published:** 2022-04-29

**Authors:** Pikuei Tu, Michaela Kotarba, Brooke Bier, Rachel Clark, Cheryl Lin

**Affiliations:** Policy and Organizational Management, Duke University, Durham, NC 27705, USA; pikuei.tu@duke.edu (P.T.); michaela.kotarba@duke.edu (M.K.); brooke.bier@duke.edu (B.B.); rachel.t.clark@duke.edu (R.C.)

**Keywords:** public health, children’s health, youth health, immunization, attitudes, health behavior, decision making, pandemic, Health Belief Model, Protection Motivation Theory

## Abstract

The COVID-19 vaccine is widely available to adolescents in the U.S.; however, vaccine hesitancy poses a threat to full coverage. The literature shows that perceived risks and the presence or lack of motivators are determinants for vaccination decisions, yet research evidence from minors is scant. This study adopted the Protection Motivation framework to identify differences in these facilitators and compare the influence of internal and external motivators among American adolescents in COVID-19 vaccine uptake. A nationwide online survey analyzed 13–17-year-old teenagers’ COVID-19 beliefs as well as present or potential reasons for accepting the vaccine. Of the 439 participants, 21.18% were not and did not plan to get vaccinated. Another 52.39% had at least one dosage, of which over three-quarters were internally motivated (whereas those unvaccinated were more likely to be externally motivated, *X*^2^ = 4.117, *p* = 0.042). In unvaccinated individuals, older adolescents reported slightly more internal motivators than younger adolescents (t = −2.023, *p* = 0.046). Internal motivation was associated with higher risk perception (r^2^ = 0.06651, *p* = 0.001), but risk perception had a stronger relationship with vaccination status (r^2^ = 0.1816, *p* < 0.001), with vaccinated individuals showing higher risk perception than those unvaccinated (mean difference = 0.42 on a scale of 1–4; t = −3.603, *p* < 0.001); the risk perception difference was even greater between hesitant and non-hesitant participants (mean difference = 0.63; t = −0.892, *p* < 0.001). The relationship was moderated by perceived knowledge, where the difference in risk perception between vaccination status was only significant for those with low perceived knowledge (f = 10.59, *p* = 0.001). Increasing awareness of disease risks and stressing internal motivators may be key to improving uptake in young people. Future research could delve deeper into risk perception formation of adolescents and why and how it differs across populations.

## 1. Introduction

After almost a year of isolation and multitudes of restrictions due to the COVID-19 pandemic, the availability of the newly developed and approved vaccines by the end of 2020 have provided a possible solution to control the spread of a life-threatening illness. However, even with the rapidly evolving and more transmissible new variants, as of March 2022, only 65.9% of the American population is fully vaccinated [1]. Despite an excess of vaccine supply in the United States, vaccine hesitancy poses an obstacle to high coverage rates. In some states, up to 30% of doses delivered remain unused [2].

Vaccine hesitancy is defined by a delay in acceptance or refusal of vaccination, existing globally and across vaccines [3,4]. The most widely recognized model of vaccine decision making is the 3C Model, outlining confidence in the vaccine, complacency towards disease risk, and convenience or access of vaccines and related services [5]. Among these constructs, perceived risk distinctly plays a key role in vaccine motivation, encompassing perceived likelihood of infection and perceived severity of disease [6]. The Protection Motivation Theory (PMT) explicates individuals’ motivation to engage in self-protecting behavior or attitude change through appraisals of threat and available coping mechanisms [7]. It can predict one’s risk perception based on the intensity of the probable threat (i.e., perceived severity of threat, perceived vulnerability to threat, or perceived benefits of not responding to the threat) and how they cope with the threat, such as their self-efficacy in undertaking protective actions and perceptions of protective behaviors’ efficacy [8]. This framework is particularly relevant for examining vaccination decision-making, in which high perceived risk and low perceived benefit of not taking action would result in behavior change [9]. Previous research has indicated a positive association between perceived risk, perceived knowledge, and adoption of protective measures against COVID-19, including vaccine acceptance [10,11,12], whereas low perceived risk correlates with lower vaccine intention and consequent exposure [13,14]. Various studies have identified PMT constructs in COVID-19-related behavior, where one is more motivated to be vaccinated when COVID-19′s severity and vulnerability perception is high, and self-efficacy is high [15,16]. These findings implicate the role of COVID-19 knowledge in PMT in both informing awareness of disease risk and one’s understanding of how to effectively engage in protective behaviors [17].

The Health Belief Model (HBM) additionally identifies that perceived benefits, presumed barriers, beliefs about disease threat, and self-efficacy may also be a function of vaccine motivation [18,19,20]. HBM posits that high perceived susceptibility, high foreseeable gain, and the presence of cue to action induce behavior [18,21]. These beliefs or perceptions are considered internal motivators, where individuals intrinsically make decisions based on a self-beneficial outcome [22]. Internal motivators have been studied in relation to the HPV vaccine, where high perceived risk and benefit encouraged intentions to receive the vaccine [23]. However, some individuals may be more heavily motivated by cues to action or in response to the choices and expectations of others, such as the recommendations of healthcare professionals or family, vaccine mandates, social pressures, or media and campaigns. The role of these external motivators has also been shown in pandemic influenza vaccine uptake among healthcare professionals [24,25].

Risk perception is a particularly important influence on adolescent health behavior, as younger populations tend to underweight negative health outcomes [26], and thus decline vaccination under the false pretense that they are at a lower risk of infection or symptom severity than adults [27], notwithstanding evidence of similar COVID incidence rates [1]. Given lagging vaccination rate in adolescents and increased variant transmissibility, understanding adolescent motivators is especially relevant [28,29]. Extant health behavior models have been applied to adolescents’ willingness to participate in other COVID-19 preventative behaviors, such as mask wearing and social distancing [30,31,32], yet there is a lack of research empirically investigating adolescents’ drivers of COVID-19 vaccine acceptance [14,27]. Among the relatively limited research on this age population, earlier studies often surveyed parents about their children and rarely directly queried adolescents to obtain their attitudes and perceptions [33]. This study aimed to fill the gap in the literature and analyzed the differences in COVID-19 vaccine decision-making across adolescent age-groups, including motivations, beliefs, opinions, and perceptions. Determining the influences of perceived risk and perceived knowledge, as well as internal compared to external motivators, on COVID-19 vaccine decisions in adolescents can help create effective and evidence-based strategies for improving uptake.

## 2. Methods

### 2.1. Participants and Data Collection

A nationwide online survey was conducted in the U.S. assessing experiences and opinions toward COVID-19 and vaccine status. Participants for this study included adolescents ranging from 13 to 17 years old who opted-in to join a panel and take surveys with prior parental permission. In determining the minimum size required to reach a representative sample at a 95% confidence level, the authors used the 2020 U.S. Census data, which indicated that there were approximately 25 million residents aged 12–17 [34] and estimated a sample of at least 384 as a target. Participant recruitment was performed through a survey panel agency to help achieve a balanced sample of gender, race, and geographic region. The agency emailed or texted (depending on individual preference) its members who met the age criterion about available surveys, which they could choose to participate in to earn points, redeemable for cash or donating to their designated non-profit organizations. The invitation only informed potential respondents of the estimated length of the survey and number of points, without disclosing the subject matter in advance to minimize self-selection bias toward certain topics or attitudes. Participation was on a first-come, first-serve basis during the allocated fielding period, contingent on reaching the pre-determined minimum quota. Data was collected in October and November 2021 from 439 participants throughout the U.S., categorized into subgroups of 13–15 and 16–17 years.

The research protocol was approved by Duke University’s Institutional Review Board, and informed consent was obtained prior to data collection. 

### 2.2. Measures

#### 2.2.1. Vaccination Status and Hesitation

Vaccination status was the primary outcome variable and is defined as “vaccinated” for those with at least one dose and “unvaccinated” for those without. To better examine attitudes, participants were separately categorized into “hesitant” and “non-hesitant” subgroups based on the question, “Do you plan to get or have you already received the COVID-19 vaccine?” The “hesitant” subgroup consisted of those who did not plan to get vaccinated or were unsure of (still deciding) whether to get vaccinated—as well as a small group of individuals that had already received one dose but were reluctant to receive a second dosage. Conversely, the “non-hesitant” subgroup consisted of fully vaccinated individuals and those planning to get their first or second dose. With vaccination status and hesitancy considered dependent variables, the impacts of the independent variables gender, age group, and race/ethnicity were considered using independent sample *t*-tests and ANOVA [35].

#### 2.2.2. Internal vs. External Motivation

**Figure 1:**
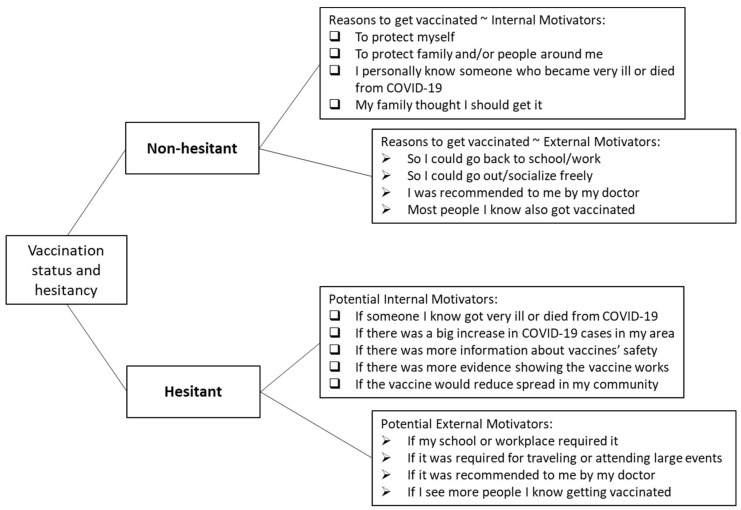
Survey query flow and motivation question items categorization.

The non-hesitant participants were asked to rank the top three reasons for getting vaccinated, whereas the hesitant participants were asked to select any listed reasons that would make them more likely to be vaccinated. Many of the items aligned with PMT’s core concept of evaluating a possible threat and coping strategy efficacy in understanding the reasons or potential motivations for individuals to undertake self-protective behavior [7]. These motivators were categorized as internal or external. Internal motivators relate to beliefs about vaccine safety and efficacy (i.e., protective behavior efficacy and benefit), the drive to avoid getting sick (i.e., the health threat individuals face), and the desire to protect loved ones; external motivators stem from requirements, recommendations, and social pressures. Figure 1 displays the query logic and answer choices with their categorizations.

For non-hesitant participants, motivation was quantified by weighting the rank of intrinsic and/or extrinsic reasons a participant chose for vaccine acceptance and totaling these values to create a composite score. Intrinsic motivations were coded on a scale of 1–3, where ranking an internal motivation as most important resulted in a 3, second most important in a 2, and third most important in a 1. Reversely, extrinsic motivations were negatively coded on a scale from −3 to −1, where ranking an external motivation as most important resulted in a −3. This method weighted factors based on how important a participant indicated them to be, creating a score ranging from −6 to 6, where a participant choosing only external factors as their top 3 motivators would receive a score of −6 and a participant choosing only internal factors would receive a score of 6.

Unvaccinated, hesitant participants were shown a list of factors, many of which were similar to those shown to non-hesitant participants (Figure 1) and asked to select any and all factors that would make them more likely to get vaccinated. These factors were not ranked, so their motivation score was unweighted and generated by assigning a −1 to each external motivation, and a +1 to each internal motivation, and totaling these values. This created a possible range of −4 to 5, where someone who selected all the motivations would receive a score of 1, someone who selected only external factors would receive a score of −4, and someone who selected only internal factors would receive a score of 5. 

These composite scores, serving as predictors, allowed researchers to use a quantitative measure of motivation for both vaccinated and unvaccinated participants. Because scoring differed based on vaccination status, the raw composite score was only used for within-group analysis of the effects of risk perception and age groups. From this information, respondents were grouped into internally and externally motivated groups as a dependent, categorical variable. Respondents with scores in the top half of the range for their vaccination status were labeled internally motivated; likewise, those with scores in the lower half were labeled externally motivated. This categorization into externally and internally motivated within vaccination status domains allowed for comparison between vaccination status domains. 

#### 2.2.3. Risk Perception

One key element of the PMT is threat appraisal, consisting of perceived probability and “noxiousness” of the threat to individuals’ health, determining one’s perceived risk [8]. Risk perception was assessed using three questions in the survey: “How likely do you think it is for you to get COVID-19 without the vaccine?”, “If you contracted COVID-19, how likely do you think you would get very sick?”, and “From what you know, how serious is the current COVID-19 situation?”. The first two questions ranged from very unlikely (1) to very likely (4). The responses for the last question ranged from “not really a concern” (1) to “very serious” (4). An individual’s risk perception score (1–4) was represented by the average of their answers to the above questions.

#### 2.2.4. Perceived Knowledge

Perceived knowledge was determined by the multiple-choice question, “Compared to others in your age group, how much do you know about the COVID-19 situation?” High perceived knowledge corresponded with answer choices, “I know a lot more about COVID-19 than most people” or “I probably know more about COVID-19 than most people”, while low perceived knowledge corresponded with answer choices, “I probably know less about COVID-19 than most people” or “I don’t really know much about COVID-19”.

### 2.3. Data Analysis

Differences in motivations between younger and older adolescents as well as vaccinated and unvaccinated adolescents were examined with chi-square tests. Then, the categories of internal and external motivation were compared using an independent sample *t*-test.

To further explore risk perception as a determinant of vaccination, independent sample *t*-tests and linear regressions were performed to compare risk perception across age groups, vaccination status, and hesitancy, and between internally motivated and externally motivated individuals [36,37]. A one-way ANOVA was run to test the association between risk perception and vaccination status, which was later compared for those with high perceived knowledge and low perceived knowledge [38]. Linear regressions were also run to test the relationships between perceived knowledge, risk perception, motivation, vaccination status, and, lastly, parent vaccination on risk perception to evaluate whether parent vaccination presented a confounding factor for the other analyses.

For each test, results were considered significant when a *p*-value ≤ 0.05 was observed. All other results were considered to show no significant difference or no significant relationship. All analyses were performed using R Studio 1.3.959 [39].

## 3. Results

### 3.1. Participant Characteristics

A total of 439 adolescents participated in the survey (sampling at a 5% margin of error at a 95% confidence level). The sample was relatively heterogeneous in terms of demographics. Participants were primarily female (53.37%) and white (57.48%). Most participants indicated that they had had at least one COVID-19 vaccine dosage (52.39%) whereas few (21.18%) were not vaccinated and did not plan to get vaccinated (Table 1). Demographic differences were present within vaccination status, where those most likely to be vaccinated were Asian (81.08% vaccinated vs. 53.60% in non-Asian) and 16–17 (56.49% versus 46.32% in 13–15). No demographic differences were found for gender.

### 3.2. Motivations across Age Groups

Overall, the most selected reasons for getting the vaccine among non-hesitant individuals were to protect their family and friends (*n* = 195, 44.3%), to protect themselves (*n* = 151, 43.1%), and to socialize or go out freely (*n* = 113, 25.6%). The factors most frequently chosen by hesitant individuals that would encourage them to get the vaccine were more evidence showing efficacy (*n* = 75, 17.1%), more information about safety (*n* = 71, 16.1%), and school mandates (*n* = 47, 10.7%). Chi-square tests run for each motivator showed that older and younger adolescents did not significantly differ in their rates of reporting for any of these motivators.

**Figure 2:**
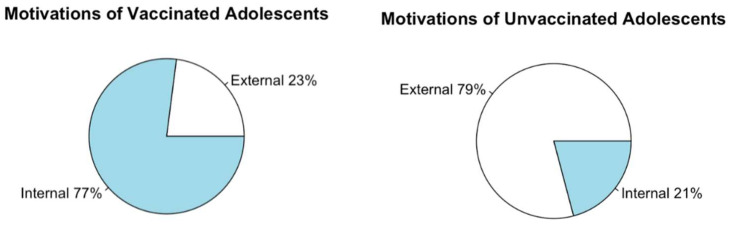
Valence of adolescent vaccination motivation by vaccination status.

The valence of motivation was related to vaccination status, with more vaccinated individuals being internally motivated than unvaccinated individuals, though at a weak significant difference (*X*^2^ = 4.117, *p*-value = 0.042). Thirty unvaccinated individuals did not select any factors as motivators, but even when this subset was removed, this relationship remained (Figure 2). For vaccinated individuals, there was no significant difference in mean motivation score between younger adolescents (mean = 1.905, SD = 3.30) and older adolescents (mean = 1.306, SD = 3.95; t = 0.881, *p*-value = 0.379), and both leaned internally motivated. For unvaccinated individuals, there was a small, borderline significant difference in motivation scores (t = −2.023, *p*-value = 0.046), with older adolescents being more internally motivated (mean = 0.418, SD = 1.22) than younger adolescents (mean = 0.085, SD = 1.01) and both scored lower than vaccinated peers on internal motivation.

### 3.3. Risk Perception

The 3-item risk perception measure had a Cronbach’s Alpha coefficient of 0.658, indicating moderate internal consistency [40,41]. Adolescents surveyed recognized the likelihood of contracting COVID-19 and the subsequent possibility of getting very sick (means = 2.64 and 2.35 on a scale of 1–4, SD = 0.982 and 0.969, respectively; correlation r = 0.294, *p*-value < 0.001), assessed the seriousness of the pandemic to be high (mean = 3.21, SD = 0.846), but age was not a determinant of perceived risk. The age groups 13–15 and 16–17 both had a mean risk perception of 2.73 out of 4 (SD = 0.58 and 0.55, respectively; t = 0.0200, *p*-value = 0.9841).

**Figure 3:**
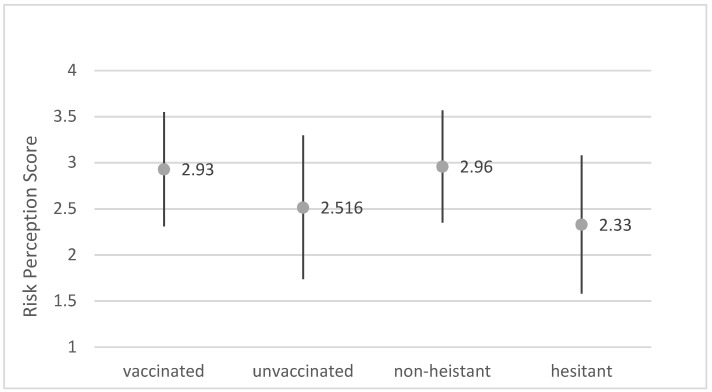
Risk perception toward COVID-19 based on vaccination status and hesitancy. Note: The dots indicate the mean risk perception score on a scale of 1–4 and the vertical lines represent the standard deviation within each subgroup. The risk perceptions between vaccinated and unvaccinated adolescents and between non-hesitant and hesitant adolescents were both significantly different (*p* < 0.05).

Vaccinated individuals had a significantly higher risk perception (mean = 2.93, SD = 0.62) than unvaccinated individuals (mean = 2.51, SD = 0.78; t = −7.064, *p*-value < 0.001). When comparing attitudes toward vaccination, there was a larger difference between hesitant participants (mean = 2.33, SD = 0.75) and non-hesitant participants (mean = 2.96, SD = 0.61; t = −8.922, *p* < 0.001) (Figure 3). The groups of adolescents overall with the highest mean risk perception scores were the non-hesitant who planned to get vaccinated but were currently unvaccinated (mean = 3.15, SD = 0.57) and the hesitant who had received one dose but were unsure about receiving a second (mean = 3.17, SD = 1). The group with the lowest mean risk perception was the unvaccinated who did not plan to get vaccinated (mean = 2.14, SD = 0.57). Finally, risk perception was higher for adolescents who reported that their parents were vaccinated than peers with unvaccinated parents (r^2^ = 0.0609, t = 3.402, *p* < 0.001).

### 3.4. Perceived Knowledge

**Figure 4:**
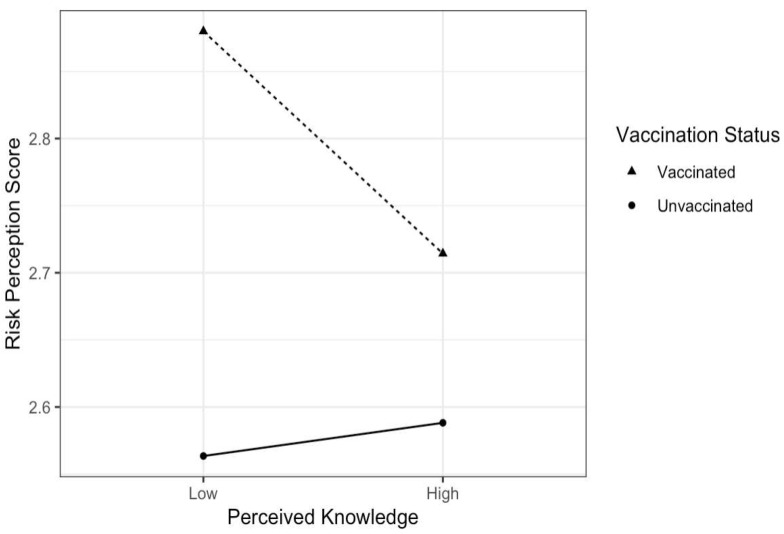
Association between adolescent risk perception and vaccination status, interaction with perceived knowledge.

Being vaccinated was significantly associated with higher risk perception (r^2^ = 0.1816, *p* < 0.001); perceived knowledge has an interaction effect on this relationship, as vaccinated individuals with low knowledge had higher risk perception than unvaccinated individuals with low perceived knowledge (Figure 4). Those with high perceived knowledge did not show a difference in risk perception score based on vaccination status (f = 10.59, *p* = 0.001). A linear regression confirmed no direct relation between perceived knowledge and risk perception (r^2^ = −0.005, *p* = 0.806).

### 3.5. Risk Perception and Motivation

With risk perception and motivation both associated with vaccine hesitancy, the authors examined the relationship between these two factors across vaccination status. Among those vaccinated, risk perception of internally motivated participants (mean = 3.107, SD = 0.5925) was significantly higher than that of externally motivated participants (mean = 2.529, SD = 0.6130; t = −3.228 *p*-value < 0.01). For the unvaccinated, there was no significant difference in risk perception scores between externally (mean = 2.358, SD = 0.777) and internally (mean = 2.273, SD = 0.7809) motivated participants (t = 1.2162, *p*-value = 0.2412).

Internal motivation was positively correlated with risk perception (r^2^ = 0.06651, *p*-value = 0.001), and those with higher internal motivation were more likely to have been vaccinated. Given that risk perception was more strongly correlated with vaccination status (r^2^ = 0.1816, *p* < 0.001), and that vaccinated individuals were more internally motivated than unvaccinated individuals (*X*^2^ = 4.117, *p*-value = 0.042, as reported above), internal motivation and higher risk perception appear to act in tandem to influence vaccination in adolescents.

The authors further compared the relative influences of risk perception and motivation on participants’ vaccine hesitancy by logistic regression. The coefficients of the two predictors were −0.982 and 0.366, respectively (both significant; r^2^ = 0.459, *p* < 0.001; Table 2), indicating that adolescents who had higher perceived risk or were more internally motivated were more likely to be non-hesitant, and that level of risk perception was a stronger indicator of vaccination decision than the valence of motivation.

## 4. Discussion

Getting adolescents vaccinated is essential in preventing the spread of COVID-19 and other preventable diseases, yet their opinions and decision-making are mostly hidden in the literature as parental responses are frequently served as a proxy in surveys. Our study reached the young population directly to compare their positions and an array of potential facilitators pertaining to vaccine acceptance. The current data showed that adolescents in the U.S. were cognizant of the pandemic’s threat and their risk perception had a greater impact on vaccine uptake and hesitancy than age or other motivators. Our findings also indicated that participants who chose to get vaccinated were more internally motivated, and those prone to be internally motivated had higher risk perception than peers who were more externally motivated. Nonetheless, unvaccinated adolescents had lower assessment of the risk regardless of their valence of motivation. A difference in motivations across age groups was only found in the unvaccinated adolescents. While perceived knowledge of COVID-19 was not directly associated with risk perception, it moderated the relationship between risk perception and vaccination decision, with high perceived knowledge youngsters reporting similar risk assessment regardless of their vaccination status.

Internal motivators and perceived-high risk groups have demonstrated greater influence of self-benefit, as opposed to social benefit, than low-risk groups for influenza and COVID-19 vaccine uptake [42,43]. From our results, over 43% of non-hesitant participants reported protecting themselves and their family as main reasons for getting the vaccine, reinforcing the role of self-efficacy in undertaking protective behavior described by PMT [7]. On the other hand, hesitant respondents may appear to focus more on the efficacy of the coping strategy (i.e., getting vaccinated), listing additional evidence on the vaccine’s safety and effectiveness as the top potential motivation; however, less than 18% of the unvaccinated selected these facilitators.

One startling finding was that about one-third of unvaccinated and unwilling respondents in our sample expressed that none of the listed motivators, even the aforementioned suggestive drivers or growing infection rate, would persuade them to get vaccinated. This alarming lack of motivation is consistent with recent poll data, where over 80% of unvaccinated adults would not change their decision if the vaccine had no side effects and/or was mandated [44]. In respect to PMT, these individuals may not vaccinate based on the low perceived cost of not getting vaccinated or low self-efficacy, rather than because of perceived threat of the virus [9]. In our study, those with the lowest perceived risk were indeed the ones stating they would not get vaccinated. Thus, public health strategies aimed to increase vaccination uptake by providing more information in the attempt to boost vaccine confidence or credibility may face challenges in persuading both the adult and younger populations.

On the other hand, individuals with a higher COVID-19 risk perception were more likely to be vaccinated. This aligns with PMT, HMB, and previous research associating threat appraisal with vaccine acceptance for both the COVID-19 and influenza vaccines, as well as other pandemic preventative behaviors [10,11,12,16,21]. This relationship is also consistent with models of adolescent risk taking, where adolescents tend to engage more in risky behaviors when perceived to be at lower risk of an outcome [31,32,45]. This study further builds on the literature by demonstrating a greater difference in risk perceptions between hesitant and non-hesitant participants than that of vaccinated and unvaccinated participants.

Furthermore, the authors found an interaction of perceived knowledge within the relationship between risk perception and vaccination status. Specifically, risk perception only differed by vaccination status for those with low perceived knowledge. Knowledge likely informs risk perception [15,46,47], but perceived knowledge was not directly correlated with risk perception. Thus, other mediating factors may have a downstream effect on vaccination status. It is important to distinguish perceived knowledge and objective knowledge as separate constructs with different effects on vaccine decisions. Some individuals may rate themselves high on knowledge, but the knowledge they hold could be inaccurate. Future research should explore the role of information accuracy, in comparison to perceived knowledge, in the relationship between vaccination intention and risk perception. For example, studies have found that educational status and level of factual knowledge, along with perceived knowledge, could sway vaccine acceptance. For this reason, targeting knowledge through vaccine education alongside strategies to increase risk perception may be useful to raise vaccine uptake.

This study had limitations. First, while the sample size met the standard margin of error at the 95% confidence level, 439 participants represent a relatively small sample, limiting the generalizability of the results. This was partially due to the restrictions in recruiting minors for research, evident in the scarcity of vaccine attitude studies directly surveying adolescents [33]. The self-report survey approach inherently has some participant selection bias, risk of social desirability bias, or errors in measurements. However, the method was appropriately chosen to assess personal beliefs and perceptions. Additionally, measuring or quantifying motivation and/or risk perception are somewhat subjective due to the lack of a standardized scale. The use of composite scores may have diluted the effects of motivations or risks that an individual perceives due to the weight given in the score. This may also explain the weak, though significant, differences observed in comparing the motivations between vaccinated and unvaccinated individuals (*p* = 0.042) and within the unvaccinated subgroup (*p* = 0.046), calling for caution in interpreting the results.

The Cronbach Alpha of the risk perception measure of 0.658 was not particularly high but satisfactory; external factors including participant characteristics and number of question items could influence the value of the coefficient [40,48]. The moderate internal consistency indicated that the items are related, but some participants responded to the three questions differently or even at the opposite ends, while others’ answers were more one-directional. For example, a vigilant individual who believes the pandemic to be somewhat serious, always wears a mask and frequently uses hand sanitizer may perceive the risk of getting infected low but the likelihood of becoming very sick high if ever contracted COVID-19.

## 5. Conclusions

Vaccine hesitancy was declared by the World Health Organization as a top 10 global health threat in 2019 [49], just before COVID-19 become a pandemic. Our study identified that risk perception has a greater impact on vaccine hesitancy than other facilitators and found vaccinated individuals to be more prone to internal, self-protective motivators than external motivators. The authors also showed support to the moderating effect of perceived knowledge relating to vaccine behavior in adolescents. Given that motivations did not show strong, consistent differences by age group and vaccination status, and that unvaccinated respondents often reported no possible motivators for future vaccine acceptance, targeting the motivations of adolescents may not yield strong results for uptake campaigns. Informing adolescents of the risks and consequences of the disease to them and those around them may be an effective strategy to increase risk perception and thus predispose adolescents to vaccine receptivity. Alternative solutions are needed specifically for adolescents, who tend to be more motivated by social rewards in decision making than their adult counterparts [45,50]. Adolescents have the power to positively influence one another, which can be harnessed to promote health preventative behaviors [51]. Future research should investigate how peer group dynamics relative to knowledge of disease and protective behaviors may encourage or dissuade vaccination, and how certain social factors can influence those who are indifferent or unmotivated [52]. Qualitative exploration on the perceptions of the unvaccinated as well as the origin of risk perception or awareness could provide context for why some conventional motivating factors were unattractive. Future studies should also account for longitudinal changes in attitudes and threat appraisal, given rapidly evolving information and events related to COVID-19 or future threats to better understand the formation of risk perception in order to effectively combat a lack of motivation.

## Figures and Tables

**Table 1:** Participant characteristics (total *n* = 439).

Variables	*n* (%) *
*Age (years)*	
13–15	177 (40.32%)
16–17	262 (59.68%)
*Self-identified Gender*	
Female	232 (52.85%)
Male	182 (41.46%)
Other/prefer not to answer	25 (5.60%)
*Self-reported Race*	
White	257 (58.54%)
Black/African American	63 (14.35%)
Hispanic/Latino	46 (10.47%)
Asian	37 (8.42%)
Native American	7 (1.59%)
Other	29 (6.63%)
*Vaccination Status*	
Unvaccinated, not planning to get vaccinated	93 (21.18%)
Unvaccinated, unsure (still undecided) whether to get vaccinated	66 (15.03%)
Unvaccinated, planning to get vaccinated	50 (11.39%)
Vaccinated, received one vaccine dose	14 (3.19%)
Vaccinated, received both vaccine doses	216 (49.20%)
*Vaccine Hesitancy*	
Non-hesitant	275 (62.64%)
Hesitant	164 (37.35%)

* Some variables do not add up to 100% because of percentage rounding.

**Table 2:** Logistic Regression of Motivation and Risk Perception on Vaccination Hesitancy ^a^.

	B	S.E.	Wald	df	Significance *p*-Value
Risk Perception ^b^	−0.982	0.128	58.641	1	<0.001
Motivation ^c^	0.366	0.087	17.729	1	<0.001
Constant	2.559	0.424	36.343	1	<0.001

^a^ Vaccination hesitancy was coded as 0 = non-hesitant and 1 = hesitant. ^b^ Risk perception was measured on a scale of 1–4, from low to high. ^c^ Motivation was categorized as internal and external.

## Data Availability

The portion of the survey data directly pertaining to this paper is available from the corresponding author for one year from the date of publication upon reasonable request with a methodically sound proposal.
